# Two clinicians for one patient, is it worth it? Patients’ perspective on receiving treatment from a pair of clinicians, in a psychiatric emergency and crisis unit

**DOI:** 10.1186/s12991-023-00446-1

**Published:** 2023-04-27

**Authors:** Caroline Dedeystère Pobelov, Orest Weber, Sonia Krenz, Yves Dorogi, Laurent Michaud

**Affiliations:** Psychiatric Liaison Service, Lausanne University Hospital and University of Lausanne, Avenue de Beaumont 23, 1011 Lausanne, Switzerland

**Keywords:** Crisis, Multidisciplinary, Setting, Joint consultation, Session, Clinicians’ role

## Abstract

**Background:**

In the field of psychiatric crisis interventions, treatment is commonly provided by multidisciplinary teams in Western countries. However, empirical data on the processes involved in this type of intervention are lacking, in particular from a patient perspective. Our study aims to gain a better understanding of the patients’ experience of a treatment setting provided by a pair of clinicians in a psychiatric emergency and crisis intervention unit. Patients’ perspective could provide a broader understanding of its advantages (or disadvantages), as well as bring new insight on elements influencing patients’ treatment adherence.

**Methods:**

We conducted 12 interviews with former patients treated by a pair of clinicians. The participants’ experience, explored with semi-structured questions on their views of the treatment setting, was analyzed by means of thematic analysis using an inductive approach.

**Results:**

The majority of participants experienced this setting as advantageous. A broader comprehension of their issues is the benefit most often expressed. A minority experienced seeing two clinicians as disadvantageous (having to talk to several clinicians at a time, change interlocutors, repeat one’s story). Participants attributed joint sessions (with both clinicians) mainly to clinical reasons and separate sessions (with one clinician at a time) mainly to logistical ones.

**Conclusions:**

This qualitative study provides first insights into patients’ experience of a setting including two clinicians providing emergency and crisis psychiatric care. The results show a perceived clinical gain of such a treatment setting for highly in crisis patients. However, further research is needed to evaluate the benefit of this setting, including the indication for joint or separate sessions as the patient’s clinical course evolves.

In the second part of the last century, outpatient mental health services have progressively developed in Western countries*.* In addition to the anti-asylum movement starting in the 1960s, cost effectiveness reasons also guided this trend towards more community and outpatient-based treatments. Various crisis intervention settings have been developed, for example mobile crisis services [1], crisis stabilization centers [2], home based crisis interventions [3], or brief therapy centers [4].

The respective roles of the different mental health professionals working in such settings have been the object of numerous articles. Some authors [5, 6] underline the importance of multidisciplinary work in a liaison psychiatry team with a clinician in charge as a supervisor. Krikorian and Fowler [7], based on their work with patients with destructive and chaotic behaviors, mention that out-of-session meetings with a supervisor enable a better understanding of the patient’s intrapsychic functioning by putting together the relational experiences of each member of the treatment team. They mention a holding function of the team enabling patients to deal with their intense emotions, limit self-destructive behaviors and engage in their treatment, a consultative/containing function enabling team members to process and interpret their relational experiences with patients, as well as an information sharing one.

More specifically concerning pairs of clinicians, based on her experience in a psychiatric emergency and crisis unit, Zucker [8] mentions logistical advantages such as treatment continuity, facilitation of contacts with the patient’s network, and security ones such as shared violence risk assessments, the possibility to reach out for help. She also mentions a decreased risk of burn-out, and that each clinician can be “the other one’s memory” considering the high patient turnover. Cludy and Botella [9], in addition to the advantages, also address constraints such as longer sessions, agenda issues, professional identity issues, etc., based on their work as a psychologist–nurse pair in an oncology department. According to some authors, based on their experience in a psychiatric emergency service [10] and a psychiatry liaison team [11], while the resident is in charge of the treatment plan, the nurse is mainly responsible for the continuity of care. Dorogi and Marguerat [11] postulate that the nurse’s caring and “holding” role is complementary to the doctor’s and enables the patient to feel sufficiently contained and supported to process and express experiences during the sessions.

Although of high clinical interest, such articles either rely on the authors’ observations [5–11], or mainly focus on the outcomes of crisis interventions [1, 3, 4, 12]. As an exception, the unpublished medical state diploma thesis of Lafaye [13] is based on a qualitative study including 14 semi-directed interviews, investigating a mobile palliative care team’s representations regarding their work in pairs of clinicians. The author mentions that working in such a setting provides support and containment, but also necessitates good communication between the clinicians, notably regarding the roles and specific skills of each professional. However, this study is based on health professionals’ and not patients’ experiences; in addition, it concerns the field of mobile palliative care.

Empirical data on the chosen setting and its pros and cons in the field of psychiatric crisis interventions are thus lacking, in particular with regard to how the patient experiences being treated by a pair of clinicians from different professions. Considering the time and financial resources implied by this model, in particular in the case of joint sessions which are often conducted in the unit where this study took place, the patients’ experience and understanding of this type of setting seems crucial. Patients’ perspective could provide a broader understanding of its advantages or disadvantages, as well as bring new insight on elements influencing patients’ treatment adherence. This study thus aims to gain a better understanding of patients’ experience of being treated by an inter-professional pair of clinicians working in a crisis intervention setting, and the dimensions which affect it.

## Methods

### Data collection

The study took place in a psychiatric emergency and crisis intervention unit in the French-speaking part of Switzerland. It offers outpatient crisis interventions with different settings, but mostly by a pair of clinicians. The treatment is based on Andreoli [14] and De Coulon’s [15] models of crisis intervention. They refer to a psychodynamic theory and imply that the patient’s current crisis resonates with intrapsychic conflicts that the person will act out in the therapeutic relationships. These may then be analyzed by the clinicians in order to understand the patient’s crisis. The crisis intervention setting includes a pair of clinicians, generally a psychiatry resident (sometimes a chief resident) and a nurse, who treat the patient during a limited time, from 3 to 8 weeks. They see the patient usually together for the first and last session. In between, they meet the patient either together or alternately, more severe symptoms being the main criteria for joint consultations.

Patient recruitment (March 2017–October 2019) was conducted by four clinicians working in the unit and part of the research team. They used a chronological list of patients having received crisis intervention in the unit. Based on the medical records, all patients whose intervention had finished during the last month at the time of recruitment, and who met the inclusion criteria (see below), received by mail an invitation to participate in the study and an information sheet. In the invitation letter, they were informed that they may be contacted by phone within a week for the study, and that they could notify their refusal to the research team by phone or by e-mail; the phone numbers and e-mail addresses from two of the four clinicians part of the research team (CD, SK, LM, YD) were included in the invitation letter. If the participant did not contact the research team members during the first week after the information sheet was sent, a research team member who was not part of the unit staff (OW) contacted him or her to explain the study more in detail, answer any questions and schedule an interview if the person was willing to participate. Participants were recruited until thematic saturation was reached.

The inclusion criteria were (i) to have received crisis intervention by an interdisciplinary treatment team (resident and nurse) for at least 3 weeks; (ii) including at least 2 sessions with both team members present; (iii) to have finished being in treatment during the last month. The exclusion criteria were to have been in treatment with a member of the research team, a lack of decision-making capacity, age below 18 years or above 65 years, insufficient languages skills in French, any medical condition incompatible with a research interview, or to have refused the audio recording.

We used an interview guide including semi-structured questions on the respondent’s experience of their treatment. This interview guide was created for the purpose of the study by the research team, based on the thesis statement. The interviews were conducted and audio taped by the same research team member who had contacted the participants and scheduled the interviews (OW). This research team member did not have access to the participant’s medical records. At the beginning of the interview, a signed consent form was obtained from the participants for the study procedures including the audio recording. All recorded interviews were transcribed and anonymized by a person who did not belong to the research team.

### Data analysis

The material was analyzed by means of thematic analysis with an inductive approach. A coding frame was developed after iterative reading of the transcripts by all investigators. To determine reliability of the coding frame, 3 of the 12 interviews were coded independently by the five authors, and their coding frames compared in group discussions. Disagreements in coding were resolved through discussions until a consensus was reached. Once the coding frame was built, each transcript was coded by a pair of investigators including one of the members of the research team (OW, YD, SK, LM) and the first author (CD).

Excerpts of the interviews were selected to exemplify the findings. No qualitative software package was used.

## Results

### Participants

37 former patients were selected based on their computerized medical records. One person called to express a refusal to participate; 36 other persons were called, out of whom 10 were not able to be reached on the phone, 8 refused to participate, 4 asked to reschedule a call and then did not answer the calls. 2 persons scheduled an interview but did not come and did not respond later to our calls.

12 former patients (7 men, 5 women) were interviewed. The age range was from 21 to 50 years old (mean: 38.3 years old). 3 patients were diagnosed with a depressive disorder, 7 with an adjustment disorder, one with PTSD (post-traumatic stress disorder), and one with an anxiety disorder, a depressive disorder as well as a personality disorder.

### Analysis

**Fig. 1:**
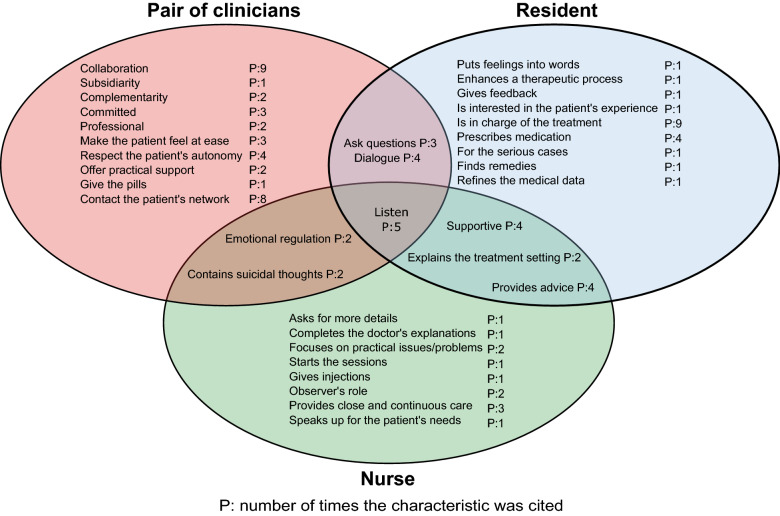
Nurse’s role defined in comparison with the doctor’s

Our analysis yielded 3 superordinated thematic fields. The first one is the participants’ understanding and evaluation of the treatment setting (see Tables 1, 2). As “treatment setting”, the participants sometimes referred to the overall setting with two mental health clinicians, sometimes to the joint sessions (with both clinicians), and sometimes to the separate sessions (with one clinician at a time). The second thematic field is the characteristics attributed to the resident, to the nurse, or to the pair of clinicians (see Fig. 1). The third thematic field (nurse’s role defined in comparison with the doctor’s) (see Table 3) is related to our finding that the participants often define the nurse’s role with regard to the doctor’s.

**Table 1:** Participants’ views on the reasons there are two clinicians and what they think of it

Logistical reasons	*P*
For the patient	
For patient convenience	1
For the institution	
Lack of time/resources	1
Internships	1

Clinical/relationship reasons	*P*
Advantages	
A more comprehensive understanding of the patient’s issues	7
Continuity of care	3
Added value of contrasting characters	2
To have a good relationship with at least one clinician	1
Positive influence of one clinician on the other, to enhance the treatment	1
Two clinicians listen better than one	1
Less stressful to talk to two clinicians	1
Possibility to adapt the setting (1 or 2 clinicians) to the patient’s illness severity	1
Disadvantages	
Uneasy to share one’s feelings and thoughts with several people	2
Disconcerting to have to interact with different people	1
Tiring to repeat things to the different clinicians	1
Lack of trust in the nursing profession, consequently in this setting	1

*P:* number of participants who referred to each item

**Table 2:** Patients’ views on the reasons for joint versus separate sessions

Joint sessions	*P*	Separate sessions	*P*
*Logistical*			
Clinicians work at the same time/do same shifts	1	Professionals’ lack of time	5
		High frequency of sessions	2
		Cost and human resources effectiveness	2
		No doctor available	2
*Clinical*			
Clinicians’ needs			
Clinicians cannot afford to make mistakes	1	Clinicians’ need to check on the patient regularly	1
Clinicians’ need to be together and confront their points of view	1		
To gain a better understanding			
Dual perspective	2	Information is collected by each clinician individually, then put together to gain a comprehensive understanding	1
Specific sessions			
Evaluation/new patient	2		
Final assessment	2		
Illness severity			
Emergency/severe symptoms	4	The nurse is enough (to treat my illness)	1

*P:* number of participants who referred to each item

**Table 3:** Nurse’s role defined in comparison with the doctor’s

Nurse’s role in comparison with the doctor’s	*P*
*Doctor’s agent*	
Under the doctor’s supervision	1
Synthesizes for the doctor	1
Conveys information to the doctor	1
Makes suggestions to the doctor	1
*Ambiguous role*	
Does not say anything	3
Whose role is not clear	3
Not qualified to listen	1
*Complementarity*	
Brings another perspective	2
Asks different questions	1
*Subsidiarity*	
Also makes decisions	1
Also gives medication	1
Has an impact on the treatment	1

*P:* number of participants who referred to each item

#### Participants’ understanding and evaluation of the treatment setting

##### Participants’ views on the reasons there are two clinicians and what they think of it

As shown in Table 1 (see below), participants mainly attribute clinical and relationship reasons to the setting, and more advantages than disadvantages. First, they value having two points of view on their difficulties, enabling a more comprehensive understanding.

“It was in order to have a more comprehensive view of the person, that is (silence) of me” (P11)

Second, several participants mention the continuity of care.

“..in times of crisis, one really needs to be cared for all the time[…] Not to hear someone say “well no, next week you cannot come because no one’s there”

(P2)

Third, participants also highlight the value of seeing two clinicians with different characters who can help them in different ways, influence each other to enhance treatment, or so that there is at least one with whom the patient can form a good connection.

“They have different characters and different ways of speaking and of saying things, and sometimes it is nice, one is a little quieter and the other pushes you a bit more, it is nice” (P2)

Fourth, one person appreciates the supportive effect provided by two clinicians who can “listen better than one”, and to whom it is less stressful to talk to than to only one professional.

Concerning the disadvantages, first, two participants mention the fact of talking to several professionals at a time.

“..but there are three people, it’s like, I don’t know, as if I was facing judges and I had to expose myself in front of several people”

(P7)

Second, one person mentions having to repeat one’s story, and third, another one expresses the fact of changing interlocutors.

“..but it’s true that to not always talk to the same person is a bit unsettling, because one.. I didn’t know if there were many things I had to say again” (P9)

##### Patients’ views on the reasons for joint versus separate sessions

As shown in Table 2 (see below), participants mainly attribute the choice of joint sessions to clinical reasons, such as specific sessions’ goals (for example first or final evaluation) or to the severity of the patient’s symptoms.

“I think that at the beginning it was more often the two of them, because it was more important and serious” (P2)

On the other hand, they mainly relate the choice of separate sessions to logistical reasons such as time and financial requirements.

" I think there were alternate sessions because, it’s a bit complicated to, to have them both there at the same time, regarding their schedules, weekly time arrangements, just that” (P12)

Two participants relate a better understanding of the patient’s case to joint sessions allowing a dual perspective during the session, while another one relates it to combining different points of view from separate sessions.

One participant considers the clinicians’ needs (to be together, because they cannot afford to make mistakes, or because they need to check on the patient regularly) as an underlying reason to choose joint or separate sessions.

“..and I had the impression that they kind of needed to be together..” (P6)

However, this finding may be considered with caution since the participant mentioned that the necessity to avoid mistakes was an explanation provided by the resident.

#### Characteristics attributed to the resident, to the nurse or to the pair of clinicians

Our data show that some characteristics are attributed to the pair of clinicians and the resident, some to the pair of clinicians and the nurse, some to the resident and the nurse, and some to the resident, the nurse and the pair of clinicians (intersections in Fig. 1).

However, some characteristics, notably those that enhance an exploration of the patient’s inner world, are mainly attributed to the resident. Whereas the ones imputed to the nurse refer more to the “here and now” of the session and what is concretely happening in the patient’s life.

“..and so on one hand there was more, well one was more objective (nurse) and the other, I don’t know if I can really say subjective but..” (P12)

Concerning their respective roles, our data suggest a clear leadership role attributed to the resident, and a more supportive and proximity role to the nurse.

“Then, indeed, concerning the decision-making and what is related, hum, it was always the doctor who had the upper hand” (P3)

“.. he (nurse) knows me better, he was seeing me the whole time” (P8)

#### Nurse’s role defined in comparison with the doctor’s

Ou﻿r data show 4 additional themes concerning the nurse’s role regarding the doctor’s: “the doctor’s agent”; “ambiguous role”, “complementarity with the doctor”, “subsidiarity with the doctor”.

One participant views the nurse’s role mainly as an agent for the doctor, while some others view it as ambiguous (Table 3).

“Thinking of it now, I said to myself why do I have to tell all this to someone who is not necessarily qualified?” (P7)

On the other hand, a few participants define the nurse’s relationship to the doctor as less hierarchical, either complementary or subsidiary.

“And who (nurse) saw things a bit differently, who interpreted things a bit differently, and that was also very positive” (P2)

## Discussion

The goal of this study was to gain an understanding of the patients’ perspective on the treatment setting provided by a pair of clinicians in a psychiatric emergency and crisis intervention unit.

Our results show that most participants consider this setting as advantageous, mainly for clinical reasons. Among the benefits, a broader understanding of the patient’s situation is the most often mentioned. Participants thus perceive a clinical value of putting together different professionals’ points of view. This is interestingly in line with De Coulon’s [15] crisis intervention model, which postulates that several clinicians who see a patient together or separately can gain a better understanding of the person’s psychic structure and functioning when sharing their observations and therapeutic experiences.

From the participants’ point of view, the choice of joint sessions is mostly guided by clinical reasons and the choice of separate sessions by logistical ones. Participants thus perceive the choice of joint sessions as more related to the patient’s state, and the choice of separate sessions as linked to exterior factors (mainly time and resource issues). Moreover, only two participants expressed feeling uneasy facing several clinicians in joint sessions, which surprised us considering the potential implications of a triadic therapeutic relationship for the patient (confusion on clinicians’ roles, less relational intimacy to address personal issues, etc.). These results may be related to unexpressed participants’ opinions in the context of this study, or to an increased tendency to regression in moments of crisis that may support the need to be taken care of by a “parental couple”. However, they may also indicate that, contrary to what we had expected, it is not such an issue for most participants to see several clinicians in a session. This could be related to a state of psychological crisis during which participants mainly need professional help and do not focus on the setting of the sessions.

These results also show that participants perceive positive effects of having two professionals taking care of them, related to the fact that they are several (two people listen better, can adapt to the patient’s needs) or to the fact that they are different (different characters, they can influence each other, establish distinct relationships with the patient). They attribute certain characteristics more specifically to the pair of clinicians (collaboration, ability to contact the patient’s network), as well as to each professional, with residents exploring the patient’s inner world and providing leadership, and nurses addressing the “here and now” and allowing proximity and containment. However, participants also attribute characteristics to both the resident and the nurse (give explanations/advice, support). In addition, when participants define the nurse’s role in comparison to the doctor’s, it is in various ways. The clinicians’ roles are thus not always clear-cut, which raises the issue of how the pairs operate, from a hierarchical and collaborative perspective (complementary or subsidiary collaboration). The small number of participants who attribute “exploratory” characteristics to the resident also supports this absence of clearly defined roles, which may be related to the lack of experience of young medical residents who make up most of the physicians in the unit where this study took place. We were in addition surprised not to find more reflections of differentiated relational experiences with the two professionals mentioned in the literature on crisis intervention [15], which could have been expressed by more polarized views on the doctor or the nurse.

Based on the outcomes of this study, we may hypothesize that when in an important state of crisis, particularly at the beginning of treatment, patients’ main concern is to get help, regardless of the number of professionals in the setting. Some may also consider several clinicians as more containing and supportive than only one. This may change as their medical state evolves. We thus recommend an on-going evaluation of the indication for an inter-professional treatment setting based on the clinical course, in particular concerning joint or separate sessions.

The patients’ understanding of the underlying reasons for the setting also seems to be a dimension that affects their experience. We can assume that if the patient attributes the setting at least partly to reasons which are directed towards his/her well-being, for example a broader understanding of the crisis issues, the patient would more positively appreciate the setting and adhere to it. This may suggest that a clear explanation about the reasons behind the clinical setting may positively enhance treatment adherence. However, we can assume that a crisis state may affect the capacity to understand such explanations. We therefore recommend that these explanations be given throughout the intervention according to an on-going assessment of the patient’s capacity to receive them.

### Strengths and limitations

This qualitative study provides first insights into patients’ experience of a setting providing emergency and crisis psychiatric care by means of a pair of clinicians. These results may support new fields of research (actual benefits/drawbacks of joint or separate sessions depending on the clinical course, usefulness and manner of explaining the setting to the patients regarding treatment adherence).

Our study also has limitations, the first one being a possible bias in the choice of participants. Considering that less signs of dissatisfaction than satisfaction with the setting emerged from the study, we may assume the participants were rather positive with regard to the care provided. Another limitation is the fact that most of the participants were either diagnosed with an adjustment disorder or a depressive disorder. Further research is needed to understand if these findings are applicable to patients presenting with other types of diagnoses, as well as to patients treated in other health care facilities than an emergency and crisis intervention unit, and by a pair of other professionals than a physician and a nurse. However, our findings also suggest that the clinicians’ professions may not always be the most important factor that affects the patient’s experience and that, particularly in a moment of crisis, their main concern is to get help, regardless of the setting, or the professionals’ roles. In addition, the small number of participants also constitutes a limitation for generalizability. Nevertheless, the primary aim of the study was to explore the patients’ perspective and gain a first in-depth understanding of their experiences with a treatment setting consisting of a pair of clinicians.

## Conclusions

These results show a perceived benefit of a psychiatric emergency and crisis intervention setting including a pair of clinicians. However, in particular considering the potential impact of a crisis state on the patients’ experience of the treatment setting, further research is needed on the advantages or disadvantages of this setting and its indications on the short or long-term (psychiatric morbidity, evolution over time, joint or separate consultations, frequency of supervision, referral to a one person setting and so on).

## Data Availability

The datasets generated and analyzed during the current study are not publicly available because public archiving of data was not explicitly authorized by the ethics committee. Nevertheless, anonymous data are available from the corresponding author on reasonable request.
